# Evaluating a Culturally Tailored Digital Storytelling Intervention to Improve Trauma Awareness in Conflict-Affected Eastern Congo: Quasi-Experimental Pilot Study

**DOI:** 10.2196/81291

**Published:** 2026-01-26

**Authors:** Achille Bapolisi, Jennifer Foucart, Déborah Kabambi, Raïssa Mirishe, Elvis Musa, Aline Ruvunangiza, Joyce Bosomi, Victor Bulabula, Marc Ilunga, Emmanuel Kajibwami, Odile Bapolisi, Arsene Daniel Nyalundja, Marie-Hélène Igega, Pacifique Mwene-batu, Philippe de Timary, Yasser Khazaal

**Affiliations:** 1 Cliniques Universitaires Saint-Luc, Department of Psychiatry, Institute of Neurosciences Faculté de Medecine UCLouvain Brussels, Brussels Belgium; 2 Faculty of Medicine Université Catholique de Bukavu Bukavu the Democratic Republic of the Congo; 3 Facultés des sciences de la motricité humaine Université Libre de Bruxelles Brussels Belgium; 4 No academic affiliation at the time of publication Bukavu the Democratic Republic of the Congo; 5 Faculté de droit Université Catholique de Bukavu Bukavu the Democratic Republic of the Congo; 6 Center for Tropical Diseases and Global Health Université Catholique de Bukavu Bukavu the Democratic Republic of the Congo; 7 Department of Clinical Sciences Liverpool School of Tropical Medicine Liverpool United Kingdom; 8 Ecole Régionale de santé publique Université Catholique de Bukavu Bukavu the Democratic Republic of the Congo; 9 Lausanne University Hospital University of Lausanne Lausanne Switzerland; 10 Department of Psychiatry and Addictology Université de Montréal Montréal, QC Canada

**Keywords:** post-traumatic stress disorder, digital media, mental health awareness, culturally adapted intervention, educational technology, psychoeducation, armed conflicts, mobile phone

## Abstract

**Background:**

Posttraumatic stress disorder (PTSD) is highly prevalent in conflict-affected regions like eastern Democratic Republic of Congo; yet, cultural stigma and lack of psychoeducation limit public understanding and help-seeking behaviors.

**Objective:**

This study evaluates the effect of a short, culturally adapted animated video on mental health perception, knowledge, and attitudes toward trauma.

**Methods:**

A community-based quasi-experimental pre-post design was implemented among 239 participants from South Kivu. The intervention involved viewing a 3-minute animated psychoeducational video portraying locally relevant PTSD symptoms and resilience strategies. Perception, knowledge, and attitude scores were measured before and after the intervention, alongside PTSD prevalence and video appreciation.

**Results:**

Out of 239, 40% (n=96) of the participants screened positively for PTSD. Post intervention, significant improvements were observed in perception (*P*=.01), knowledge (*P*<.001), and attitudes (*P*=.001) toward trauma. Appreciation was high; 82% (n= 195) expressed empathy for the characters, and 74% (n= 176) were likely to share the video. Linear regression showed that having PTSD symptoms (β coefficient=3.29, SE=1.09; *P*=.003), years of education (β coefficient=0.54, SE=0.08; *P*<.001), empathy toward the portrayed situations (β coefficient=5.07, SE=0.56; *P*<.001), perceived acquisition of new knowledge (β coefficient=2.58, SE=0.59; *P*<.001) and willingness to share the video (β coefficient=1.75, SE=0.50; *P*=.001) predicted stronger positive effect. A multiple linear regression including all predictors revealed that PTSD symptoms (β coefficient=1.93, SE=0.90; *P*=.03), years of education (β coefficient=0.47, SE=0.07; *P*<.001), empathy toward the portrayed situations (β coefficient=3.50, SE=0.55; *P*<.001), and willingness to share the video (β coefficient=1.75, SE=0.50; *P*=.001) remained significant predictors of video impact. Age and perceived acquisition of new knowledge were not significant in the multivariate model. This model accounted for 44.6% of the variance in video impact scores (*R*^2^=0.446, *F*_6,231_=30.99, *P*<.001).

**Conclusions:**

This study highlights the effectiveness of culturally grounded, low-cost digital media for improving mental health literacy in postconflict settings. Video-based tools may serve as scalable components of trauma-informed care and public health communication in low-resource, high-need areas.

## Introduction

Raising awareness and educating the public about mental health is a major challenge, both in the fields of public health and psychotraumatology [1]. Better knowledge of mental disorders contributes not only to their prevention and treatment but also to the promotion of psychological well-being and the rehabilitation of those affected. Ideally, awareness strategies should rely on accessible, low-cost, widely shareable, and culturally appropriate means of communication.

Mental health disorders are increasingly recognized as a major contributor to morbidity and mortality worldwide, with an expected growing burden in low- and middle-income countries. Mental health conditions are among the top 10 causes of years lived with disability globally. In 2021, they made up around 15.6%-17.2% of all years lived with disability [2]. Among these disorders, posttraumatic stress disorder (PTSD) is drawing increasing attention from clinicians, researchers, and public health policymakers due to its rising prevalence, its psychological and relational impact, and its numerous psychiatric and somatic comorbidities [3]. The emergence of PTSD is closely linked to the growing frequency of traumatic events worldwide: armed conflicts, acts of terrorism, natural disasters exacerbated by climate change, road accidents, and more. One international study estimates that 70% of the global population has been exposed to at least 1 potentially traumatic event during their lifetime [4].

In response to this reality, movements such as trauma-informed interventions [5] and trauma-informed care [6] have emerged to promote greater recognition of trauma in both society and health care. These approaches aim to legitimize psychological suffering, raise awareness of trauma’s impacts, and encourage clinical responses that are empathetic and appropriate. Despite these advances, many regions of the world, particularly in sub-Saharan Africa, remain largely unaffected by such awareness-raising initiatives and the integration of trauma into mental health policies [7]. Moreover, in these regions, mental health remains a marginal concern due to limited resource allocation and low levels of mental health literacy, both of which contribute to reduced accessibility and acceptability of mental health services [8].

Cultural differences largely explain the low levels of information, recognition, and acceptance of psychiatric care in these contexts [9]. This is especially concerning given that these same regions are often the most exposed to conflict. The Democratic Republic of Congo, marked by nearly 3 decades of armed violence resulting in more than 4 million deaths [10], is a stark illustration of this situation. In the most affected areas, PTSD prevalence reaches up to 40% [11]. The resurgence of conflict in February 2025 has further exacerbated the situation, profoundly impacting local populations. Yet, the mental health response is hindered by several obstacles, such as lack of information and awareness, stigma, reliance on alternative belief systems, and a shortage of specialized resources [12-14]. Patients with psychological disturbances often seek help from traditional healers before turning to psychologists or psychiatrists. Moreover, the historical divide between modern medical approaches and traditional practices has deepened a gap that remains difficult to bridge. Although the Democratic Republic of the Congo remains one of the poorest countries in the world [15], with over 52% of its population living on less than US $2.15 per day [16], it is nonetheless experiencing the effects of global digitalization, particularly in major urban areas where smartphone use and internet access are rapidly increasing [17]. This rapid digitalization offers new opportunities to extend communication and improve mental health literacy among the population. Recent digital health research underscores the importance of culturally sensitive design in improving user engagement and intervention outcomes across diverse settings [18].

To address these challenges, we developed an innovative approach that combines the opportunities offered by digitalization and the growing use of social media in the Democratic Republic of Congo with local cultural representations. We designed a short video featuring characters that portray common manifestations of PTSD while suggesting possible paths to resilience. Short video–based interventions have recently shown strong potential to improve mental health literacy and reduce stigma in community settings [19]. Digital storytelling was chosen over other digital interventions because it combines narrative, emotion, and cultural resonance—3 elements shown to strengthen engagement and knowledge retention in health communication. In eastern Congo, where literacy levels vary widely, and stigma surrounding mental illness remains strong, stories conveyed through relatable characters and familiar cultural symbols can communicate complex psychological concepts more effectively than text-based or purely informational approaches. The narrative format allows individuals to recognize their own experiences within the story, promoting empathy, normalization of psychological distress, and reduction of stigma. Moreover, the use of animation and local languages increases accessibility across literacy levels and cultural groups. This approach aligns with growing evidence that culturally grounded digital storytelling can enhance mental health literacy and foster behavioral intentions to seek help in low-resource and conflict-affected contexts [20-22].

This pilot study was designed to explore the potential of such a video-based intervention to enhance mental health perceptions, knowledge, and attitudes in South Kivu, a region heavily affected by armed violence. This quasi-experimental pilot study was conducted in South Kivu, a region heavily affected by armed conflict, to assess the intervention’s potential to improve trauma-related perceptions, knowledge, and attitudes. The study also sought to provide preliminary insights into the acceptability, feasibility, and short-term impact of this low-cost, scalable psychoeducational tool. We hypothesized that viewing the culturally adapted video would result in (1) improved perceptions and knowledge about psychological trauma and (2) more positive attitudes toward professional help-seeking and social support compared with preintervention measures.

## Methods

### Video Capsule Development Process

An initial team composed of YK, JF, PdT, and AB outlined the main features of the video capsule. It was designed to be no longer than 3 minutes and aimed primarily to provide trauma-focused psychoeducation in a culturally adapted format. The duration was deliberately limited to less than 3 minutes, as shorter videos have been shown to maximize viewer engagement, comprehension, and completion rates in health education contexts, particularly among audiences with varying literacy levels [23-27]. In addition, concise videos are easier to share through popular social media and mobile messaging platforms commonly used in the Democratic Republic of Congo, facilitating wider community dissemination [17].

AB subsequently conducted interviews with 1 psychiatrist and 2 psychologists with extensive clinical experience in the region, to identify the most commonly reported symptoms and the most frequently mobilized resilience strategies. Reported symptoms included nightmares, flashbacks, sadness, palpitations, and difficulties with concentration. Additionally, some interviews highlighted a local representation of trauma as a form of spiritual attack or curse affecting several members within a community.

In terms of resilience strategies, community and family support, physical activity, and psychomedical follow-up were cited as the most helpful resources. Based on this input, AB drafted an initial script featuring a diverse set of characters intended to reflect the pluralistic nature of society.

This script was shared with local illustrators (MI and EK) familiar with cultural and contextual specificities. A collaborative staging process followed, allowing for detailed discussions of each visual and narrative element (Multimedia Appendix 1).

Once the final version of the video capsule was completed, AB presented it to various groups, including students (n=50), merchants (n=29), and colleagues in psychiatry and psychology (n=5), to assess its clarity, intelligibility, and relevance. To enhance accessibility, the video used simplified language and clear visual storytelling, allowing key messages to be understood without reliance on text. Narration was provided in 5 local languages—Swahili, Lingala, Tshiluba, Kikongo, and French—to support comprehension among participants with low literacy or mild communication difficulties. These adaptations ensured inclusivity and cultural relevance across diverse audiences, and versions with on-screen captions are currently being developed to further improve accessibility.

**Figure 1:**
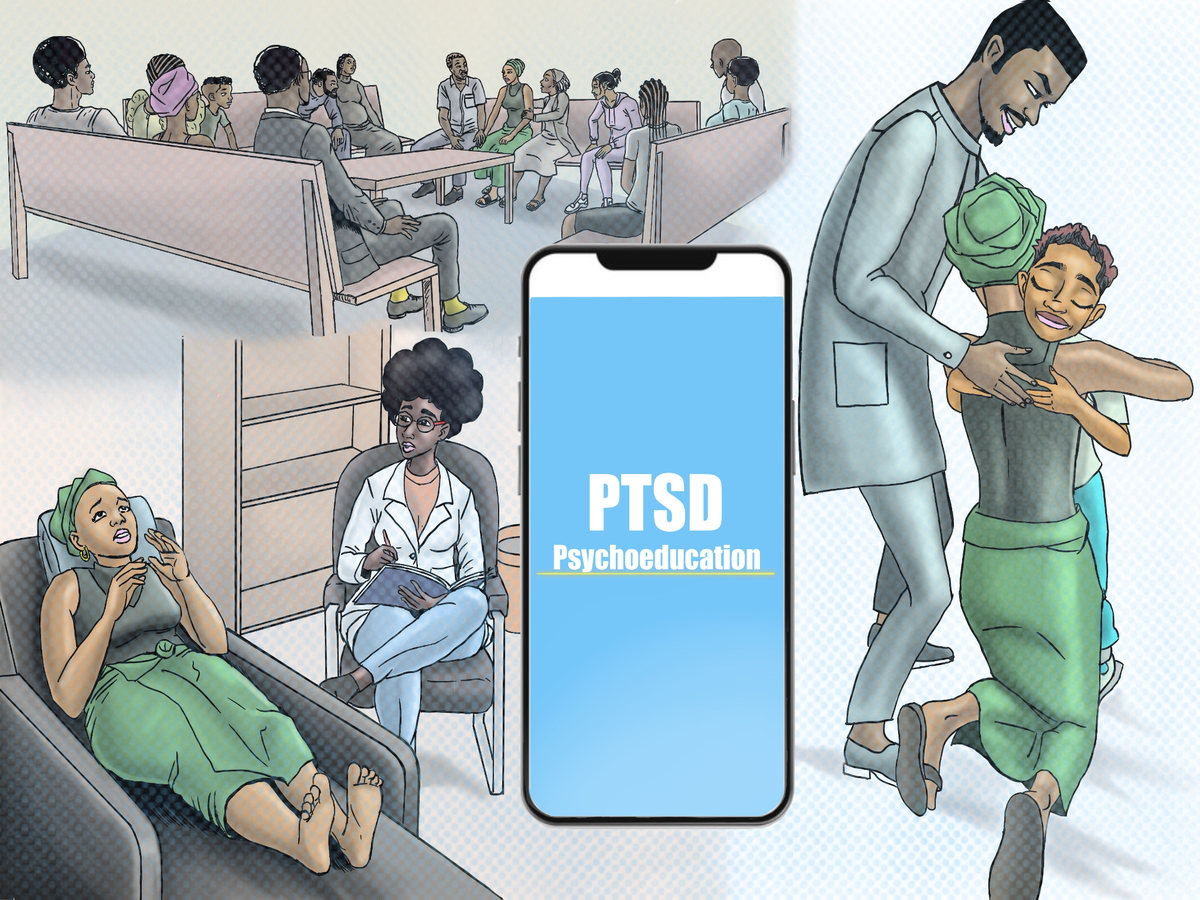
Illustrative scenes from the culturally adapted animated video used in the intervention.

Some illustrative scenes from the culturally adapted animated video used in the intervention are presented in Figure 1. The animation portrays locally recognizable experiences and emotional responses to trauma, including fear, distress, and social withdrawal, as well as supportive interactions with health care professionals and community members. Characters were designed to reflect the cultural and social diversity of eastern Congo, aiming to foster empathy, identification, and understanding of PTSD and pathways to resilience.

### Study Design and Setting

We conducted a community-based quasi-experimental pre-post study to evaluate changes in perception, knowledge, and attitude related to mental health following the viewing of an educational video capsule.

Measurements were taken immediately before and after the intervention. The study used an immediate postintervention assessment to capture the short-term effects of the video on perceptions, knowledge, and attitudes toward psychological trauma. This approach was chosen for both methodological and contextual reasons. Given the security volatility in eastern Congo, the risk of population displacement, and limited communication infrastructure, a follow-up assessment was not feasible within the study’s timeframe. Immediate postassessment ensured that the same participants could be evaluated under consistent conditions while minimizing attrition bias. Moreover, as a pilot study, the primary objective was to determine the intervention’s short-term acceptability and feasibility rather than long-term behavioral change. Nevertheless, we acknowledge that perceptions, knowledge, and attitudes are constructs that influence behavioral intentions and actions over time, and future studies are planned to include longitudinal follow-ups to assess retention and potential behavioral outcomes.

The study took place in the eastern region of the Democratic Republic of Congo, specifically in the city of Bukavu and the rural surrounding areas of Nyantende, Idjwi, Ciriri, and Kalehe. These regions have historically been affected by repeated episodes of armed conflict. Data collection was conducted between December 2024 and January 2025, during a period of relative calm that preceded the escalation of conflict in February 2025.

### Inclusion and Exclusion Criteria

All adults aged 18 years and older who had been residing in Bukavu, Idjwi, Kalehe, Ciriri, or Nyatende for at least 6 months were eligible to participate.

Individuals who were severely ill, exhibited severe mental disorders, or had significant communication impairments (eg, hearing or speech difficulties and inability to provide informed consent) were excluded from the study.

### Sample Size Determination

The sample size was calculated to detect a statistically significant change in perception of psychotrauma, measured as a continuous variable, using a paired-sample *t* test. With a 2-tailed α of .05, 80% power, a moderate effect size (Cohen *d*=0.5), and assuming a pre-post correlation of 0.5, the required minimum sample size was estimated at 34 participants, using G*Power software. However, our final sample consisted of 239 participants, which not only met the requirement for the primary outcome but also allowed for broader analyses, including the estimation of the prevalence of PTSD.

### Sampling Procedure

A multistage sampling technique was used to ensure both representativeness and logistical feasibility. South Kivu province is administratively divided into several territories and urban communes that vary substantially in population density and exposure to conflict. In the first stage, 5 zones were selected at random using a lottery method from the list of health zones in the province. In the second stage, within each selected zone, districts and rural sectors were stratified according to their population size and accessibility. From these, 1 urban district and 4 rural sectors were randomly selected.

Within each selected area, approximately 30% of neighborhoods were then randomly chosen. This proportion was determined a priori as a balance between representativeness and operational feasibility, given constraints related to time, security, and available field personnel. The 30% threshold was therefore fixed across sites to maintain comparability of sampling intensity, while the specific neighborhoods were selected randomly within each site based on administrative population listings provided by local authorities.

Finally, within each selected neighborhood, the target number of participants was proportionally allocated according to estimated population size, and households were approached consecutively until quotas were met. Because this was an exploratory pilot study rather than a population-weighted survey, clustering effects were not incorporated in the sample size calculation. However, data collection procedures were standardized across sites to minimize intercluster variability, and potential design effects were evaluated during analysis through comparison of site-level means and variances. No significant clustering effect was observed. The intervention was delivered individually to each participant using smartphones operated by trained research assistants. Participants viewed the video once in a quiet setting immediately before completing the postintervention assessment. This personalized format was chosen to ensure that all participants could clearly see and hear the content. All research assistants followed a standardized protocol to maintain fidelity across sites, including identical video files, playback settings, and instructions. Data collection procedures were harmonized through a detailed manual and a 3-day training workshop focused on consistent delivery, participant guidance, and confidentiality. Site-level consistency was verified during analysis, and no significant variation in intervention effects was observed between locations, in coherence with the standardization of delivery and data collection procedures.

### Perception, Knowledge, and Attitudes Toward Trauma

The perception questionnaire was adapted from the studies on perceptions in mental health [28], and included 4 items. The knowledge and attitude sections were adapted from the study on trauma-informed care [29], comprising 7 items on knowledge and 6 items on attitude.

Perception was assessed using an inverse Likert scale ranging from 0 (strongly agree) to 4 (strongly disagree), while knowledge and attitudes were assessed using a standard Likert scale ranging from 0 (strongly disagree) to 4 (strongly agree). Individual scores were summed to produce total scores for each domain.

To ensure cultural and contextual accuracy, the questionnaires were translated into French and Swahili, then independently back-translated into English by bilingual experts. The translated versions were reviewed by a panel of local psychiatrists and psychologists to verify conceptual equivalence and cultural appropriateness of terms and examples. Before data collection, the instruments were pilot tested among 20 adults from Bukavu and Idjwi to assess clarity, comprehensibility, and cultural relevance. Feedback from this pilot phase led to minor wording adjustments to reflect local idioms of distress and expressions of emotional states. Internal consistency reliability for the final scales was satisfactory (Cronbach α=0.81 for perception, 0.86 for knowledge, and 0.84 for attitudes).

The effect score of the video was calculated as the difference between the total score obtained after viewing and the total score obtained before viewing, by summing all item scores at each time point.

### Appreciation of the Video Capsule

We also assessed participants’ appreciation of the video capsule using 3 questions that evaluated (1) empathy toward the characters and situations portrayed, (2) perceived acquisition of new knowledge, and (3) willingness to share the video. Responses were measured on a Likert scale ranging from *not at all* to *extremely*. We calculated the number and percentage of participants selecting each response level.

### Psychological Assessment

We measured past traumatic events and PTSD through the Post-traumatic Diagnostic Scale - French adaptation (PDS-F), along with a Stressful Events Scale [30], a detailed scale assessing the types and magnitude of a wide variety of traumatic events as well as PTSD symptoms. This scale showed good psychometric values in African populations [31] and has a validated French version [30]. The PDS-F is interpreted with a severity score ranging from 0 to 51 obtained by adding up the responses of items. For the positive screening of PTSD to be made, we considered the cutoff for moderate to severe symptoms, a rating of >20 [32].

### Data Collection Procedures and Quality Control

Data were collected by 5 medical students. The principal investigator (AB) trained the medical students over 3 days, emphasizing the theoretical and practical aspects of all questions in the questionnaire, informed consent, and participant confidentiality.

### Ethical Considerations

Ethical approval was obtained from the Catholic University of Bukavu Ethics committee (UCB/CIES/NC/033/253). We obtained written informed consent from participants, and we ensured their privacy and confidentiality. Participants were then offered, after the completion of the evaluation sessions, with the main investigator, where needed, and those with psychological disturbances were advised to attend counseling sessions in the psychiatric clinic of the hospital. No financial or material compensation was provided for participation.

### Data Analysis

We analyzed data using Stata (version 13; StataCorp) to perform descriptive and inferential analysis. Qualitative variables were described in terms of frequencies and percentages, while continuous variables were described in terms of means and SDs. Pearson chi-square and the Student *t* test were used to compare characteristics of patients for categorical and continuous variables, respectively.

We conducted bivariate and multiple linear regression analyses to explore associations between demographic, psychological, and appreciation-related variables and the overall effect score on perceptions, knowledge, and attitudes toward trauma. Variables showing a bivariate association with the outcome at *P*≤.05 were entered into the multivariate model. Model building followed a stepwise strategy: theoretically relevant variables (age, sex, education level, and PTSD symptom severity) were entered first to control for potential confounding, followed by appreciation variables (empathy, perceived acquisition of new knowledge, and willingness to share the video) reflecting emotional engagement and behavioral intention. The inclusion of these predictors was guided by the theory of planned behavior and previous evidence linking affective engagement and trauma exposure to psychoeducational outcomes. Model fit and multicollinearity were assessed using the coefficient of determination (*R*^2^), adjusted *R*^2^, *F* statistics, and variance inflation factors (VIFs; all VIF<2), confirming appropriate specification and absence of collinearity.

We conducted bivariate and multiple linear regression analyses to explore associations between demographic, psychological, and appreciation-related variables and the overall effect score on perceptions, knowledge, and attitudes toward trauma. Variables showing a bivariate association with the outcome at *P*≤.05 were entered into the multivariate model. Model building followed a stepwise strategy: theoretically relevant variables (age, sex, education level, and PTSD symptom severity) were entered first to control for potential confounding, followed by appreciation variables (empathy, perceived acquisition of new knowledge, and willingness to share the video) reflecting emotional engagement and behavioral intention. The inclusion of these predictors was guided by the theory of planned behavior and previous evidence linking affective engagement and trauma exposure to psychoeducational outcomes. Empathy enhances perspective taking and prosocial intentions, which are associated with greater openness toward trauma-related information [33-37]. Likewise, perceived self-relevance and social relevance are robust predictors of video sharing intentions, as individuals are more likely to share content they find personally meaningful or beneficial to their social network [38,39]. Emotional resonance—such as empathy, hope, or identification with the scenario—fosters authenticity and social connection, motivating sharing behavior and reinforcing learning through peer support [40]. Trust in the source or creator also predicts sharing behavior, with higher information and science literacy associated with more discerning and health-promoting dissemination [41,42]. In line with the theory of planned behavior, knowledge contributes to behavioral change by shaping attitudes, perceived behavioral control, and subjective norms, which in turn influence intentions and actions [42,43]. The inclusion of age, sex, and education level as predictors is further supported by extensive evidence showing their association with posttraumatic stress symptom severity and psychoeducational outcomes [44-46]. Model fit and multicollinearity were assessed using *R*^2^, adjusted *R*^2^, *F* statistics, and VIFs (all VIF<2), confirming appropriate specification and absence of collinearity.

## Results

### Sociodemographic Characteristics and PTSD Prevalence

A total of 239 participants were enrolled, comprising 133 (56%) women and 106 (44%) men, with a mean age of 34 (SD 14) years. Of the total, 147 (62%) participants were married, and 79 (33%) were unemployed. Regarding education, 86 (36%) had completed university education, and the mean number of years of schooling was 10 (SD 7) years. There were significant sex differences in marital status, profession, and education level (*P*<.05). The overall prevalence of probable PTSD (positive screening for PTSD) was 40%, with no significant difference between sex (*P*=.22; Table 1).

**Table 1:** Sociodemographic characteristics and posttraumatic stress disorder prevalence (N=239).

Variable		Total	Female	Male	*P* value
Sex, n (%)		239 (100)	133 (56)	106 (44)	—^a^
Age (y), mean (SD)		34 (14)	34 (13)	34 (14)	.92
**Marital status, n (%)**					.002
	Married	147 (62)	91 (68)	56 (53)	
	Single	78 (33)	31 (23)	47 (44)	
	Separated or divorced	6 (2)	6 (5)	0 (0)	
	Widower	8 (3)	5 (4)	3 (3)	
**Occupation, n (%)**					
	Liberal activity	52 (22)	33 (25)	19 (18)	<.001
	State official	19 (8)	5 (4)	14 (13)	.29
	Humanitarian worker	13 (5)	3 (2)	10 (9)	.005
	Unemployed	79 (33)	48 (36)	31 (29)	<.001
	Student	32 (13)	11 (8)	21 (20)	<.001
	Others	52 (23)	33 (25)	19 (18)	.21
**Education level, n (%)**					
	Less than primary	46 (19)	36 (27)	10 (9)	—
	Primary	30 (13)	23 (17)	7 (7)	.001
	Secondary	77 (32)	45 (34)	32 (30)	.06
	University	86 (36)	29 (22)	57 (54)	.29
Years of education, mean (SD)		10 (7)	8 (7)	12 (6)	<.001
PTSD^b^ prevalence, n (%)		96 (40)	58 (44)	38 (36)	.22

^a^Not applicable.

^b^PTSD: posttraumatic stress disorder.

### Perception, Knowledge, and Attitudes Toward Trauma

Following the video intervention, participants showed significant improvements in perception, knowledge, and attitude scores, respectively (Table 2). The mean perception score increased from 11.8 (SD 2.5) to 12.4 (SD 2.4; *P*=.01). Several perception items, such as the belief that “talking about one’s suffering is useless,” improved significantly (*P*=.01).

**Table 2:** Participant responses to video content (N=239).

Item	Likert scale response, n (%)				
	Not at all	Very little	Moderately	A lot	Extremely
To what extent did you feel empathy toward the people or situations portrayed in the video?	4 (1.7)	8 (3.3)	32 (13.4)	105 (43.9)	90 (37.7)
To what extent did the video provide you with new information or knowledge?	5 (2.1)	10 (4.2)	32 (13.4)	99 (41.4)	93 (38.9)
How likely are you to share this video with others in your network?	8 (3.3)	9 (3.8)	46 (19.2)	90 (37.7)	86 (36)

Knowledge scores improved markedly, rising from 19.3 (SD 5.5) to 21.2 (SD 3.9; *P*<.001). Significant changes were observed in items relating to trauma’s effect on mental and physical health and recognition of trauma symptoms (eg, nightmares and palpitations).

Similarly, attitudes toward trauma improved, with the total score increasing from 16.2 (SD 4.3) to 17.5 (SD 4.7; *P*=.001). Key items, such as belief in the possibility of recovery and the need for professional support, showed significant positive shifts.

Effect sizes were calculated for each domain to complement significance testing. The intervention produced small to moderate improvements in perception (Cohen *d*=0.26), knowledge (Cohen *d*=0.38), and attitude (Cohen *d*=0.28). Site-level analyses were also performed to examine potential regional variation in outcomes; no significant differences were observed across sites, suggesting consistent intervention effects throughout the different study areas.

A detailed version of these results, including all item-level data, can be consulted in **Multimedia Appendix 2**.

### Appreciation of the Video Capsule

Participants’ feedback on the video was positive (Table 2). Out of 239 participants, 195 (82%) felt empathy “a lot” or “extremely” toward the portrayed situations, 192 (80%) reported having acquired new knowledge, and 176 (74%) stated that they were likely or very likely to share the video.

### Predictors of Video Effect

Simple linear regression analyses revealed several significant predictors of higher video effect scores. Strongest associations were observed for video appreciations: higher levels of empathy toward the portrayed situations (β coefficient=5.07, SE=0.56, *t*_237_=9.07; *P*<.001), perceived acquisition of new knowledge (β coefficient=2.58, SE=0.59, *t*_237_=4.36; *P*<.001), and willingness to share the video (β coefficient=3.30, SE=0.53, *t*_237_=6.23; *P*<.001) were all significantly associated with greater video effect. Among demographic and clinical variables, years of education showed a strong positive effect (β coefficient=0.54, SE=0.08, 95% CI 0.39-0.69, *R*^2^=0.171; *P*<.001), while PTSD symptoms were also positively associated with video effect (β coefficient=3.29, SE=1.09, 95% CI 1.13-5.44, *R*^2^=0.037; *P*=.003). In contrast, age was inversely associated with video effect (β coefficient=–0.11, SE=0.04, 95% CI –0.19 to –0.03, *R*^2^=0.031; *P*=.007).

A multiple linear regression including all predictors revealed that PTSD symptoms (β coefficient=1.93, SE=0.90, *t*_231_=2.14; *P*=.03), years of education (β coefficient=0.47, SE=0.07, *t*_231_=6.69; *P*<.001), empathy toward the portrayed situations (β coefficient=3.50, SE=0.55, *t*_231_=6.33; *P*<.001), and willingness to share the video (β coefficient=1.75, SE=0.50, *t*_231_=3.47; *P*=.001) remained significant predictors of video effect. Age and perceived acquisition of new knowledge were not significant in the multivariate model. This model accounted for 44.6% of the variance in video effect scores (*R*^2^=0.446, *F*_6,231_=30.99; *P*<.001; Table 3).

**Table 3:** Multiple linear regression predicting video effect (N=238). Model fit: F6,231=30.99, R2=0.446, adjusted R2=0.432; root-mean-square error=6.60; *P*<.001.

Variable	β coefficient (SE)	95% CI	*t* test (*df*)	*P* value
Age	–0.003 (0.033)	−0.07 to 0.06	–0.10 (231)	.92
Years of education	0.47 (0.07)	0.33 to 0.61	6.69 (231)	<.001
PTSD^a^ score	1.93 (0.90)	0.15 to 3.71	2.14 (231)	.03
Empathy toward the portrayed situations	3.50 (0.55)	2.41 to 4.59	6.33 (231)	<.001
Perceived acquisition of new knowledge	0.70 (0.55)	−0.38 to 1.78	1.28 (231)	.20
Willingness to share the video	1.75 (0.50)	0.76 to 2.74	3.47 (231)	.001
Constant	27.35 (2.55)	22.32 to 32.38	10.72 (231)	<.001

^a^PTSD: posttraumatic stress disorder.

## Discussion

### Principal Findings

This study demonstrated that a short, culturally adapted animated video significantly improved participants’ perceptions, knowledge, and attitudes toward psychological trauma in conflict-affected eastern Congo. After viewing the video, mean scores for perception, knowledge, and attitudes increased markedly, with large effect sizes and high participant engagement—over 80% of viewers reported empathy for the characters and 74% indicated willingness to share the video. Regression analyses further revealed that higher education, empathy toward the portrayed situations, and the presence of PTSD symptoms were significant predictors of stronger intervention effects. Together, these findings indicate that culturally grounded, low-cost digital storytelling can effectively enhance mental health literacy and reduce stigma in low-resource, trauma-affected settings.

Our findings align with and extend evidence from video-based and multimedia psychoeducational interventions. For example, the effect of online multimedia psychoeducational interventions on the resilience and perceived stress of hospitalized patients with COVID‑19 found significant improvements in resilience and reductions in perceived stress after a brief online multimedia intervention [47]. Our findings are also consistent with evidence from trauma-specific psychoeducational interventions, which similarly produced moderate improvements in PTSD, depression, and bonding outcomes among pregnant women with trauma histories, demonstrating the potential of brief, structured psychoeducation to enhance psychological well-being in vulnerable populations [48]. In our study, a culturally tailored, 3-minute animated video delivered individually, the small-to-moderate effect sizes we observed suggest that even very brief interventions can produce meaningful changes in mental-health literacy in conflict-affected settings.

The mechanisms underlying the observed changes likely stem from enhanced cognitive and emotional engagement achieved through a culturally grounded narrative. By portraying locally recognizable experiences and characters, the video fostered empathy, identification, and reflection—key processes known to mediate learning and attitude change in health communication [49,50]. Delivering the intervention individually via smartphones, with multilingual narration and culturally adapted visuals, further improved accessibility, attention, and comprehension, thereby supporting effective knowledge transfer and stigma reduction.

Low-cost, scalable digital interventions are increasingly recognized as practical strategies for improving health literacy and reducing stigma in resource-limited settings [51]. In this study, the creation of an animated video on PTSD, based on symptoms and experiences most frequently described by Congolese clinicians and patients, offered a familiar and emotionally engaging medium for psychoeducation. The animation, developed by local artists, embodied the region’s cultural and linguistic diversity and avoided overly medicalized or Western frameworks that may alienate local audiences [8,52,53]. Such a design, consistent with human factors principles, enhances usability, accessibility, and long-term engagement with digital mental health content, even in low-resource contexts [54].

The changes observed in perception, knowledge, and attitudes suggest that video capsules like this could be an effective tool for psychoeducation and for reducing stigma associated with mental health issues. Previous studies have shown that individuals suffering from psychological disorders are often heavily stigmatized in these populations, where seeking mental health care is perceived as shameful [55,56]. Survivors of rape and torture are also frequently stigmatized due to perceived social shame and dishonor [57,58]. The video was therefore designed to legitimize the suffering resulting from trauma and to encourage understanding and social support.

One justified concern in war-affected contexts is the risk of retraumatization or vicarious trauma [6,59,60]. Empathy elicited by trauma-related video content is consistently associated with increased emotional distress, including heightened anxiety, depressive symptoms, and physiological stress responses, such as elevated heart rate and cortisol, particularly in individuals with high emotional contagion or affective empathy [34,37,61-63]. This issue was central to the development of the video, and efforts were made to avoid overly distressing images. The emotional impact of the content was carefully reviewed throughout the process. In addition, as described in our methodology, ethical safeguards were in place to recommend appropriate care for participants exhibiting signs of trauma. In addition to illustrating the psychological consequences of trauma, the video also depicts multiple pathways to resilience—individual coping efforts, family and community support, and access to psychomedical assistance—reflecting an integrated, culturally grounded approach to recovery.

Our findings also show that participants with PTSD reported higher empathy toward the characters and experienced a more pronounced positive impact on their perceptions, knowledge, and attitudes after viewing the video. One of the most striking findings of our study is the relatively high prevalence of positive screening for PTSD (40%). While this figure may appear elevated compared with other studies [64-66], it aligns closely with previous research conducted in the conflict-affected regions of eastern Congo [11]. Furthermore, the recent deterioration in regional security—marked by the resurgence of a rebel group that ultimately forced us to halt data collection—likely heightened the expression of traumatic symptoms within the population.

Finally, our study shows that the video’s positive effect was particularly evident among young, educated individuals and those showing posttraumatic symptoms. This finding supports the targeted use of such interventions among youth, students, and individuals at risk of PTSD. These results point to the potential for implementing video-based awareness tools in schools, universities, and health care settings, contributing meaningfully to trauma-informed interventions and trauma-informed care, with both clinical and public health implications.

Taken together, our findings suggest that culturally contextualized video capsules can foster positive changes in how psychological trauma is perceived, understood, and addressed in war-affected regions. Our findings align with the theory of planned behavior [67], which posits that intention is a proximal predictor of behavior. The positive shifts in attitudes toward professional help, reduced stigma, and increased endorsement of social support suggest an enhanced behavioral intention to seek care. While actual care-seeking behavior was not directly measured, our results imply a potential increase in future help-seeking behaviors.

### Limitations

While this study provides valuable insights into the potential of culturally adapted video interventions to improve mental health awareness in conflict-affected regions, several limitations should be acknowledged. First, the quasi-experimental pre-post design without a control group limits causal inference. Although improvements in perception, knowledge, and attitudes were observed after viewing the video, we cannot fully rule out the influence of external factors or testing effects. Second, the follow-up was immediate and did not assess the sustainability of changes over time. Longer-term follow-up would be necessary to determine whether improvements in knowledge and attitudes persist and translate into behavioral change or increased care-seeking. Third, the self-report nature of the measures may have introduced bias, including social desirability effects, particularly in postintervention responses. Additionally, while the instruments used were adapted from validated tools, further validation in this specific cultural context would strengthen the robustness of the findings. Although internal consistency coefficients were good, these indices primarily reflect internal reliability rather than the full spectrum of psychometric validity. Future research should therefore include confirmatory factor analyses and convergent or discriminant validity assessments to ensure that the constructs measured retain their theoretical structure and meaning in this cultural setting. Fourth, although efforts were made to ensure linguistic and cultural appropriateness, the diverse ethnic and linguistic backgrounds of participants in South Kivu mean that certain nuances may not have been fully captured by a single video version. Future studies could explore more tailored adaptations by subgroup and by level of instruction. Finally, the study sample, although geographically diverse, may not be representative of the broader Congolese population or other conflict-affected regions. Moreover, individuals with severe mental illness or communication impairments were excluded, possibly underestimating the intervention’s reach and effect among the most vulnerable populations.

### Conclusion

This study highlights the potential of culturally adapted, multimedia interventions to improve mental health literacy in conflict-affected regions. By integrating local representations of trauma with evidence-based psychoeducation, the video capsule successfully enhanced participants’ perceptions, knowledge, and attitudes toward psychological trauma. The positive reception of the intervention, particularly among individuals with PTSD symptoms and those with higher education levels, underscores the value of contextually sensitive and accessible approaches to mental health promotion. In settings like eastern Democratic Republic of Congo, where traditional stigma, limited resources, and ongoing violence hinder mental health care, such tools may represent a promising, scalable strategy to foster trauma-informed awareness, reduce stigma, and support pathways to resilience. Continued investment in culturally grounded public health communication and further evaluation in large-scale and long-term impact will be essential for strengthening mental health systems in similar contexts.

## Data Availability

The datasets generated or analyzed during this study are not publicly available due to participant confidentiality, but are available from the corresponding author upon reasonable request.

## Appendix

Multimedia Appendix 1 Sample video capsule (English version) used in the intervention. The 3-minute animated clip illustrates locally relevant manifestations of psychological trauma (such as nightmares, avoidance, and emotional distress) and highlights culturally meaningful resilience strategies, including community and family support, physical activity, and professional help-seeking. The video was produced collaboratively with Congolese illustrators and mental health professionals to ensure cultural and linguistic appropriateness.

Multimedia Appendix 2 UntitlTable : Perception, Knowledge, and Attitude Scores Before and After Visualizationed.

## Abbreviations

PDS-F: Post-traumatic Diagnostic Scale – French adaptation
PTSD: posttraumatic stress disorder
VIF: variance inflation factor
