# Supplementary material for: Evaluating a Culturally Tailored Digital Storytelling Intervention to Improve Trauma Awareness in Conflict-Affected Eastern Congo: Quasi-Experimental Pilot Study
Source: JMIR Ment Health. 2026 Jan 26;13:e81291. doi: 10.2196/81291 (PMC12887569; doi:10.2196/81291)
Supplement: Multimedia Appendix 2 [file mental_v13i1e81291_app2.doc]

**Multimedia Appendix2:**

**Table : Perception, Knowledge, and Attitude Scores Before and After Visualization**

| Variable | Item | Score Before Visualization (Mean ± SD) | Score After Visualization (Mean ± SD) | p-value |
| --- | --- | --- | --- | --- |
| Perception | After a trauma, only the weak are affected | 3.4 (1.0) | 3.5 (1.0) | .45 |
|  | After a trauma, talking about one's suffering is useless | 3.1 (1.1) | 3.4 (1.0) | .01 |
|  | People who suffer from mental problems after a trauma were already mentally ill to begin with | 2.3 (1.4) | 2.2 (1.4) | .64 |
|  | After a trauma, talking about one's suffering is shameful | 3.2 (1.2) | 3.3 (1.2) | .70 |
|  | Total perception | 11.8 (2.5) | 12.4 (2.4) | .01 |
| Knowledge | Exposure to trauma is common | 3.6 (1.1) | 2.9 (1.0) | .003 |
|  | Trauma affects mental well-being | 2.9 (1.0) | 3.2 (0.7) | < .001 |
|  | Trauma affects physical well-being | 3.0 (0.9) | 3.2 (0.8) | .003 |
|  | Link between psychiatric disorders and past trauma | 2.8 (0.9) | 3.1 (0.9) | .01 |
|  | Abnormal behaviors may be linked to trauma | 2.7 (1.1) | 2.9 (1.1) | .08 |
|  | Recurrent nightmares as sign of trauma | 2.6 (1.2) | 2.9 (1.0) | < .001 |
|  | Recurrent palpitations as sign of trauma | 2.6 (1.2) | 3.0 (0.9) | < .001 |
|  | Total knowledge | 19.3 (5.5) | 21.2 (3.9) | < .001 |
| Attitude | Possible to recover from trauma | 2.6 (1.0) | 2.9 (1.0) | < .001 |
|  | Healing paths vary by individual | 2.1 (1.3) | 2.3 (1.3) | .09 |
|  | Traumatized people deserve support | 2.9 (1.1) | 3.0 (1.1) | .31 |
|  | Consultation with health professionals | 3.0 (0.9) | 3.2 (0.9) | .004 |
|  | Traumatized people can decide what they need | 2.3 (1.3) | 2.6 (1.4) | .02 |
|  | People should be informed about health consequences | 3.2 (0.9) | 3.3 (0.9) | .07 |
|  | Total attitude | 16.2 (4.3) | 17.5 (4.7) | .001 |
|  | Global video impact | 47.2 (8.4) | 51.1 (8.8) | < .001 |
